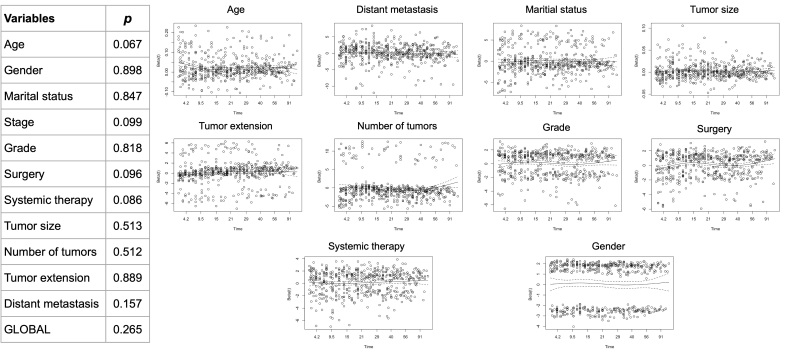# Machine learning-based individualized survival prediction model for prognosis in osteosarcoma: Data from the SEER database

**DOI:** 10.1097/MD.0000000000039582

**Published:** 2024-09-27

**Authors:** Ping Cao, Yixin Dun, Xi Xiang, Daqing Wang, Weiyi Cheng, Lizhao Yan, Hongjing Li

**Affiliations:** a Department of Orthopedic, The Frist Affiliated Hospital of Dalian Medical University, Dalian, China; b Department of Orthopedic, Tianyou Hospital, Wuhan University of Science and Technology, Wuhan, China; c Department of Emergency General Surgery, Union Hospital, Tongji Medical College, Huazhong University of Science and Technology, Wuhan, China; d Department of Hand Surgery, Union Hospital, Tongji Medical College, Huazhong University of Science and Technology, Wuhan, China.

**Keywords:** deep learning, DeepSurv, machine learning, osteosarcoma, survival analysis

## Abstract

Patient outcomes of osteosarcoma vary because of tumor heterogeneity and treatment strategies. This study aimed to compare the performance of multiple machine learning (ML) models with the traditional Cox proportional hazards (CoxPH) model in predicting prognosis and explored the potential of ML models in clinical decision-making. From 2000 to 2018, 1243 patients with osteosarcoma were collected from the Surveillance, Epidemiology, and End Results (SEER) database. Three ML methods were chosen for model development (DeepSurv, neural multi-task logistic regression [NMTLR]) and random survival forest [RSF]) and compared them with the traditional CoxPH model and TNM staging systems. 871 samples were used for model training, and the rest were used for model validation. The models’ overall performance and predictive accuracy for 3- and 5-year survival were assessed by several metrics, including the concordance index (C-index), the Integrated Brier Score (IBS), receiver operating characteristic curves (ROC), area under the ROC curves (AUC), calibration curves, and decision curve analysis. The efficacy of personalized recommendations by ML models was evaluated by the survival curves. The performance was highest in the DeepSurv model (C-index, 0.77; IBS, 0.14; 3-year AUC, 0.80; 5-year AUC, 0.78) compared with other methods (C-index, 0.73–0.74; IBS, 0.16–0.17; 3-year AUC, 0.73–0.78; 5-year AUC, 0.72–0.78). There are also significant differences in survival outcomes between patients who align with the treatment option recommended by the DeepSurv model and those who do not (hazard ratio, 1.88; *P* < .05). The DeepSurv model is available in an approachable web app format at https://survivalofosteosarcoma.streamlit.app/. We developed ML models capable of accurately predicting the survival of osteosarcoma, which can provide useful information for decision-making regarding the appropriate treatment.

## 1. Introduction

Osteosarcoma is the most common primary bone malignancy in adolescents and young adults, typically affecting those between the ages of 10 and 20.^[1]^ Osteosarcoma is characterized by a high recurrence rate, aggressive behaviors, and early metastasis.^[2]^ Even in the absence of obvious metastases, most of these patients had subclinical metastases at diagnosis.^[3,4]^ Before the era of effective chemotherapy, 80 to 90 per cent of patients experienced tumor recurrence after surgery.^[5]^

With the use of chemotherapy and advances in surgical techniques in the past decades, about two-thirds of patients with non-metastatic extremity osteosarcoma can survive long-term, and up to 50% of patients with limited lung metastases can be cured.^[6–8]^ Postoperative chemotherapy was initially used in the 1970s, and 5-year survival rates rose from less than 20% to 40% to 60%. The concept of induction or neoadjuvant chemotherapy combined with limb salvage surgery emerged. It takes time to make custom-made metal implants, so chemotherapy is often given while waiting for radical surgery.^[9]^ Another advantage of preoperative chemotherapy is that it can be used as an in vivo drug test to determine the drug sensitivity of an individual tumor and to customize postoperative treatment.^[10,11]^ Consequently, the current standard perioperative chemotherapy for osteosarcoma comprises MAP (methotrexate, doxorubicin, cisplatin) before and after surgery, and the sequence of chemotherapy and surgery is still under investigation.^[11,12]^ Surgery is also advancing alongside chemotherapy. While tumor eradication remains the main goal, doctors now favor more conservative procedures aimed towards preserving function. Limb salvage surgery, instead of amputation, is now available for most patients, especially those who have used preoperative chemotherapy.^[13]^

Accurate patient prognosis prediction is important for the oncologist team when determining the best course of therapy for the patients, as well as for patients to plan their limited resources.^[14]^ Nomogram is the most commonly used survival analysis method and is widely used in clinical,^[15,16]^ genetic,^[17]^ and imaging data^[16]^ to predict the prognosis of osteosarcoma. Nomogram is based on the CoxPH (Cox Proportional Hazard) model, which is a multivariable linear model to fit the correlation between survival time and predicted variables. The CoxPH model has 2 fundamental assumptions: the log-hazard of an individual is a linear function of their covariates; the hazards are proportionate to the individual, which means that the relative hazard remains constant over time.^[18]^ However, the linear nature of the CoxPH model potentially neglects complex and possibly nonlinear relationships between features, especially in the face of patients’ complex clinical characteristics and high dimensional data.^[14,19]^ Meanwhile, it is also too strict to assume that the variable’s effect on survival does not change over time.^[18]^

With the development of artificial intelligence, the application of machine learning (ML) in survival analysis also shows obvious advantages.^[20,21]^ Currently, various ML models have been applied to survival analysis, such as DeepSurv,^[22]^ neural multi-task logistic regression (NMTLR),^[23]^ random survival forest (RSF),^[24]^ Cox-nnet,^[25]^ DeepHit,^[26]^ etc. Among them, DeepSurv is a representative work to extend deep learning to CoxPH models by replacing linear predictors with multi-layer neural networks, giving the model the ability to fit nonlinear relationships between features. Many studies have proved that the DeepSurv method is superior to the traditional CoxPH model,^[27,28]^ and the superiority of DeepSurv becomes more obvious with the improvement of data quality.^[22]^ The NMTLR model uses a neural network to directly parameterize multi-task logistic regression, which directly computes the survival function on the predetermined time grid after discretizing survival time.^[23,29]^ The RSF model is an ensemble model that calculates the average value of the cumulative hazards and is not constrained by the proportionality assumption.^[24,29]^ In addition, due to their nonlinear nature, ML models can fit the relevant interaction patterns between features.^[22]^ Therefore, when a model is used in clinical decision-making, the ML model can provide personalized treatment recommendations for each patient, unlike the CoxPH model, which provides the same treatment recommendation for all patients based on hazard ratio (HR) value.^[22,30]^ These 3 models represent different modeling approaches and have shown excellent performance in previous studies. Due to the nonlinear nature of the data, we aim to explore the potential of nonlinear models like DeepSurv, NMTLR and RSF in osteosarcoma prognosis prediction.

With the diversification of treatment options and the increased volume of data, the application of ML in prognosis prediction and clinical decision-making of osteosarcoma is promising. In the current study, we developed and validated 3 machine learning-based survival prediction models (DeepSurv, NMTLR, and RSF) using a comprehensive dataset from the SEER database. Our models were compared with traditional methods (CoxPH and TNM staging) and demonstrated superior performance in terms of prediction accuracy and clinical utility. We also demonstrated the potential of these ML models to provide individualized treatment recommendations, improving decision-making in clinical settings. Finally, we developed a user-friendly web application based on the optimal model, facilitating easy access and usage by clinicians for prognostic assessment and treatment planning.

## 2. Materials and methods

### 2.1. Data source

All patients with osteosarcoma included in this study were selected from the SEER “18 Regs Research Plus Nov 2020 Sub (2000–2018 varying) data set (http://seer.cancer.gov). Approximately 28% of all cancer patients in the United States are represented in the SEER database, which is a cancer-specific database in the United States that contains the morbidity, mortality, and disease data of millions of individuals with malignant tumors. After completing the SEER Research Data Agreement form and emailing it in, we were granted access to the database.

### 2.2. Study population

**Figure 1.**
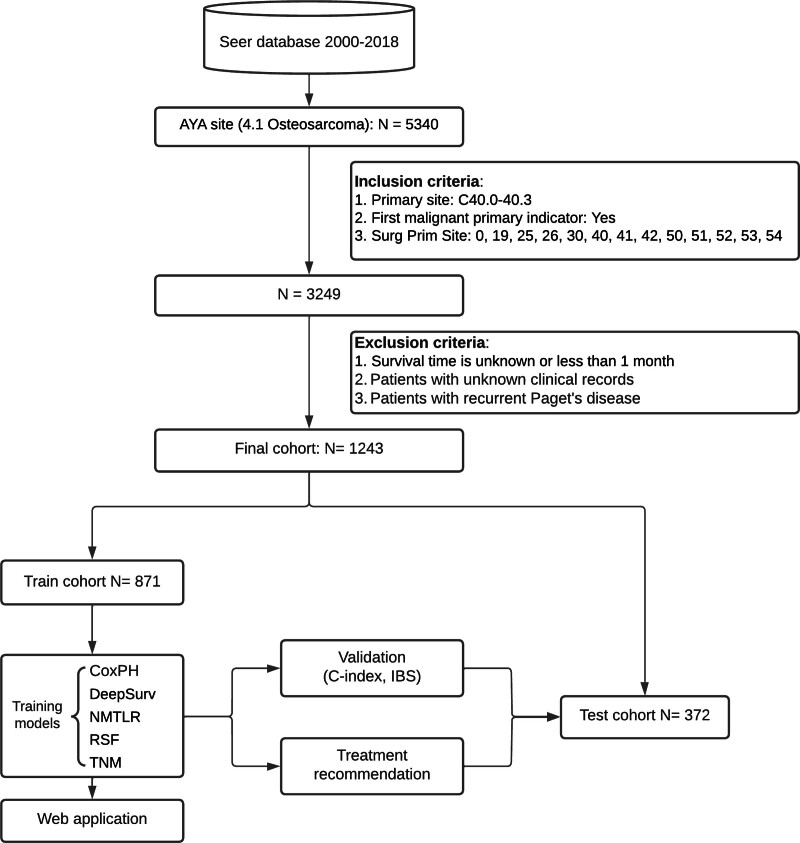
Study profile and analysis pipeline.

We extracted data from patients newly diagnosed with osteosarcoma between January 1, 2004, and December 31, 2015, with the primary site code C40.0–40.3. Baseline and clinical variables were extracted, including gender, age, race, marital status, tumor size, number of tumors, histologic grade, primary site, tumor extension, distant metastasis, tumor site, tumor stage, surgical type, and systemic therapy. For osteosarcoma in the SEER database, systematic treatment refers to chemotherapy. The inclusion criteria were used for the selection of cases: Patients with a confirmed diagnosis of osteosarcoma. Long and short bones as the primary site (Primary site = C40.0, C40.2). osteosarcoma was identified as the first primary tumor. Several exclusion criteria were also applied: Patients with incomplete prognosis information. Patients with recurrent Paget’s disease. Survival time of less than 1 month. Figure 1 depicts a flowchart of the comprehensive selection procedure.

### 2.3. Model development

Both univariate and multivariate Cox regression was utilized for feature selection. Statistical significance was defined as a *P* value less than .05, and all comparisons were made at the 95% confidence level. To rule out any collinear characteristics, correlation tests were also performed between features. Three ML-based models were chosen for model development, including DeepSurv, neural network multitask logistic regression (NMLTR), and RSF. Besides, a multivariate CoxPH and a univariate TNM model were built as the baseline method for evaluation. In a 7:3 random split, the dataset was used for training and for validating models, respectively. Training data was used to train the ML algorithms that consider time to event with the overall survival (OS) as the outcome. In model training, the strategies of cross-validation and hyperparameter random search are used to optimize the model parameters. The detailed process of model building and hyperparameter search space can be found in our repository on GitHub: https://github.com/WHUH-ML/Osteosarcoma.

### 2.4. Verification of Cox proportional hazards model assumptions

To ensure the validity of the CoxPH model, we conducted tests to verify the proportional hazards assumption. This was done using Schoenfeld residuals, which assess whether the residuals from the model are independent of time. Specifically, for each covariate, we plotted the Schoenfeld residuals against time and tested for non-zero slope, which would indicate a violation of the proportional hazards assumption.

### 2.5. Model evaluation and validation

The Harrell concordance index(C-index),^[31]^ which measures the correlation between predicted survival risks and actual survival times, was used to assess the reliability of the models. The C-index ranges from 0.5 for a completely random prediction to 1.0 for a model with perfect predictive ability. A Brier score was also presented; it indicates the mean square difference between the observed status and the predicted survival probabilities of a patient and ranges between 0 and 1, with a lower value indicating a better result. Besides, the Integrated Brier Score (IBS) offered a comprehensive evaluation of the performance of the model over all of the accessible time periods.^[32]^ Calibration for the 3- and 5-year OS, which compared the predicted survival with the observed survival, was performed with a calibration curve. Receiver operating characteristic (ROC) curves were mapped, and the area under ROC curves (AUC) value was utilized to evaluate the accuracy of the prediction of the survival probability at a particular time. Decision curve analysis (DCA) was performed to determine the clinical usefulness of a model by calculating the net benefits at different threshold probabilities.^[33]^

### 2.6. Risk stratification

All the patients in the test dataset were categorized into low-risk and high-risk groups, respectively, based on the risk score that was obtained by the models to further validate the practicability of our prediction model. The cutoff values were determined based on the models’ respective mean values for the risk score.

### 2.7. Treatment recommendation

When multiple treatment options are available, a model recommends a treatment option that has the minimum overall predicted risk when all other features are held constant. However, the treatment of patients has already occurred in our study, so we divided the patients who are consistent with the treatment plan recommended by the model into the “Recommendation” group and those patients who are inconsistent with the treatment plan recommended by the model into the “Anti-Recommendation” group. Due to the linear relationship between the traditional CoxPH fitted features, it recommends the same treatment for all patients based on HR values. In contrast, the ML model offers nonlinearity advantages, enabling it to make individualized treatment recommendations based on interaction patterns between treatment-related and other baseline characteristics.^[22]^ Finally, a log-rank test was used to check the differences between the 2 groups in survival probability. In this study, we use models to make treatment recommendations of systematic therapy (chemotherapy) options for osteosarcoma patients and compared them with the actual treatments to verify the performance.

### 2.8. Feature importance

To evaluate the importance of features to a model, we replaced each feature value with random numbers sequentially and took the c-index reduction of the model after replacement as the evaluation measure.^[30]^

### 2.9. Statistical analysis

All continuous variables in the clinical data are displayed as the mean standard deviation and compared with unpaired 2-sided *t* tests. Categorical variables are described as frequencies and percentages compared with the chi-square test between groups. Data pre-processing and plotting were completed using (version 4.0.5; http://www.r-project.org) and R studio (version 1.4.1717; https://www.rstudio.com). The PySurvival package of Python (version 3.6.8, https://www.python.org/) was used to create ML models.

## 3. Results

### 3.1. Basic characteristics

A total of 1243 patients with osteosarcoma met our inclusion criteria. The baseline information of the patients at the time of enrollment is shown in Table 1. Their mean age was 24.37 ± 18.21 years, and 56.9% of these were male. 871 patients were assigned to the training cohort, and 372 patients were assigned to the test cohort. The mean OS was 60.99 ± 40.28 months in the training group and 61.85 ± 42.11 in the test group. there was no significant difference for each feature between the training and test cohorts (*P* > .05) (Table 1).

**Table 1:** Characteristic distribution of data in training sets and test sets.

	Level	Overall	Train	Test	*P* value
n		1243	871	372	
Age (mean (SD))		24.37 (18.21)	24.38 (18.25)	24.35 (18.14)	.978
Gender (%)	Female	536 (43.1)	366 (42.0)	170 (45.7)	.256
	Male	707 (56.9)	505 (58.0)	202 (54.3)	
Marital status (%)	Not married	1029 (82.8)	723 (83.0)	306 (82.3)	.811
	Married	214 (17.2)	148 (17.0)	66 (17.7)	
Stage (%)	IA	63 (5.1)	44 (5.1)	19 (5.1)	.792
	IB	53 (4.3)	34 (3.8)	19 (5.4)	
	IIA	292 (23.5)	205 (23.4)	87 (23.7)	
	IIB	505 (40.6)	342 (41.7)	163 (38.2)	
	III	28 (2.3)	21 (2.3)	7 (2.2)	
	IVA	157 (12.6)	112 (12.1)	45 (14.0)	
	IVB	145 (11.7)	112 (11.7)	33 (11.6)	
Grade (%)	Well differentiated	50 (4.3)	33 (4.0)	17 (4.9)	.580
	Moderately differentiated	76 (6.5)	51 (6.2)	25 (7.1)	
	Poorly differentiated	358 (30.6)	260 (31.7)	98 (28.0)	
	Undifferentiated	687 (58.7)	477 (58.1)	210 (60.0)	
Surgery (%)	None	107 (8.7)	72 (8.3)	35 (9.4)	.572
	Amputation	281 (22.8)	196 (22.7)	85 (22.9)	
	Local destruction or excision	110 (8.9)	83 (9.6)	27 (7.3)	
	Radical excision with limb salvage	736 (59.6)	512 (59.3)	224 (60.4)	
Systemic therapy (%)	Not	246 (19.8)	172 (19.7)	74 (19.9)	.434
	Before surgery	538 (43.3)	370 (42.5)	168 (45.2)	
	After surgery	319 (25.7)	234 (26.9)	85 (22.8)	
	Both before and after surgery	140 (11.3)	95 (11.9)	45(12.1)	
Tumor size (mean (SD))		107.03 (56.63)	107.23 (56.23)	106.54 (57.65)	.850
Number of tumors (%)	1	1141 (91.8)	798 (91.6)	343 (92.2)	.817
	>1	102 (8.2)	73 (8.4)	29 (7.8)	
Tumor extension (%)	No break in periosteum	240 (19.5)	165 (19.2)	75 (20.3)	.745
	Extension beyond periosteum	930 (75.5)	651 (75.6)	279 (75.4)	
	Further extension	61 (5.0)	45 (5.2)	16 (4.3)	
Distant metastasis (%)	None	955 (77.1)	674 (77.6)	281 (75.7)	.558
	Lung only	164 (13.2)	109 (12.6)	55 (14.8)	
	Other distant organ	120 (9.7)	85 (9.8)	35 (9.4)	
Survival months (mean (SD))		61.24 (40.82)	60.99 (40.28)	61.85 (42.11)	.733
Status (%)	Alive	744 (59.9)	518 (59.5)	226 (60.8)	.720
	Dead	499 (40.1)	353 (40.5)	146 (39.2)	

### 3.2. Feature selection

**Figure 2.**
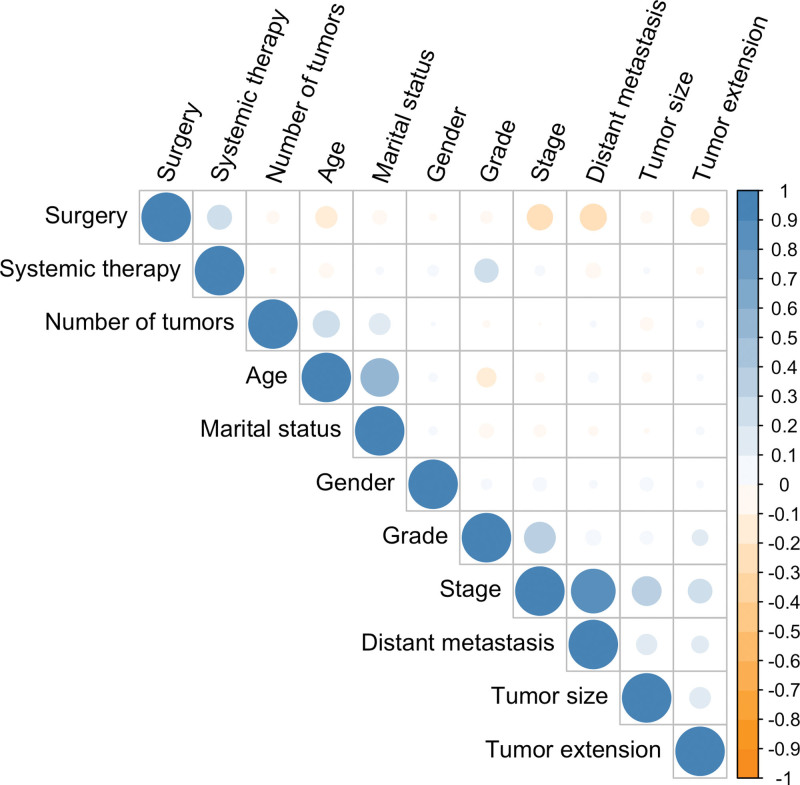
Correlation coefficients for each pair of variables in the data set. The estimated correlation values are distributed within the range of −1 to +1. They are represented by color depth, with a number closer to either end value implying a stronger negative correlation or positive correlation.

Univariate and multivariate Cox regression analyses were performed for all data. As shown in Table 2, 11 significant factors (age, gender, marital status, stage, grade, surgery, systemic therapy, tumor size, number of tumors, tumor extension, and distant metastasis) were selected by cox regression analysis. In the correlation analysis, the stage feature was excluded because of its high collinearity with other features (Fig. 2). Ultimately, a total of 10 features were defined as independent prognostic factors and included in the development of the final model.

**Table 2:** Patient demographic, disease, treatment characteristics, and Cox regression analysis.

Characteristic	Overall	Univariate Cox			Multivariate Cox		
	N = 1243	HR	95% CI	*P* value	HR	95% CI	*P* value
Age	24 (18)	1.02	1.02, 1.03	**<.001**	1.03	1.02, 1.03	**<.001**
Gender				**.019**			.28
Female	536 (43)	—	—		—	—	
Male	707 (57)	1.24	1.03, 1.48		1.12	0.91, 1.39	
Marital status				**<.001**			>.99
Not married	1029 (83)	—	—		—	—	
Married	214 (17)	1.62	1.31, 2.00		1.00	0.74, 1.36	
Race				.16			.25
White	922 (74)	—	—		—	—	
Black	204 (16)	1.24	0.99, 1.56		1.18	0.91, 1.53	
Other	115 (9.3)	1.14	0.85, 1.54		1.26	0.90, 1.77	
Unknown	2						
Primary site				.47			.17
Long bones	1214 (98)	—	—		—	—	
Short bones	29 (2.3)	0.80	0.43, 1.50		0.59	0.27, 1.31	
Stage				**<.001**			**.005**
IA	63 (5.1)	—	—		—	—	
IB	53 (4.3)	1.65	0.37, 7.36		1.11	0.25, 5.03	
IIA	292 (23)	6.36	2.01, 20.1		6.68	1.41, 31.5	
IIB	505 (41)	8.76	2.80, 27.4		8.09	1.73, 38.0	
III	28 (2.3)	22.4	6.66, 75.4		11.4	2.22, 58.3	
IVA	157 (13)	22.9	7.25, 72.2		44.3	5.85, 336	
IVB	145 (12)	36.9	11.7, 116		25.9	4.53, 148	
Grade				**<.001**			.95
Well differentiated	50 (4.3)	—	—		—	—	
Moderately differentiated	76 (6.5)	1.92	0.61, 6.02		0.93	0.24, 3.52	
Poorly differentiated	358 (31)	6.60	2.44, 17.8		1.05	0.24, 4.71	
Undifferentiated	687 (59)	6.16	2.30, 16.5		0.99	0.22, 4.41	
Unknown	72						
Surgery				**<.001**			**<.001**
None	107 (8.7)	—	—		—	—	
Amputation	281 (23)	0.33	0.25, 0.44		0.61	0.37, 1.00	
Local destruction or excision	110 (8.9)	0.24	0.16, 0.34		0.50	0.29, 0.85	
Radical excision with limb salvage	736 (60)	0.18	0.14, 0.24		0.40	0.25, 0.66	
Unknown	9						
Systemic therapy				**<.001**			.34
Not	246 (20)	–	–		–	–	
Before surgery	538 (43)	0.54	0.43, 0.68		0.75	0.49, 1.14	
After surgery	319 (26)	0.67	0.52, 0.86		0.90	0.59, 1.38	
Both before and after surgery	140 (11)	0.83	0.61, 1.12		0.81	0.52, 1.26	
Tumor size	107 (57)	1.01	1.00, 1.01	**<.001**	1.00	1.00, 1.00	**.006**
Unknown	69						
Number of tumors				**.001**			.98
1	1141 (92)	–	–		–	–	
> 1	102 (8.2)	1.61	1.22, 2.12		0.99	0.71, 1.40	
Tumor extension				**<.001**			**.021**
No break in periosteum	240 (19)	–	–		–	–	
Extension beyond periosteum	930 (76)	1.71	1.31, 2.24		1.35	1.00, 1.82	
Further extension	61 (5.0)	4.07	2.77, 5.99		2.12	1.22, 3.68	
Unknown	12						
Distant metastasis				**<.001**			.38
None	955 (77)	–	–		–	–	
Lung only	164 (13)	3.13	2.50, 3.91		0.55	0.15, 2.08	
Other distant organ	120 (9.7)	5.23	4.14, 6.60		1.11	0.46, 2.64	
Unknown	4						
Survival months	61 (41)						
Status							
Alive	744 (60)						
Dead	499 (40)						

Mean (SD); n (%).

CI = confidence interval, HR = hazard ratio.

### 3.3. Verification of cox proportional hazards model assumptions

To ensure the validity of the CoxPH model, we conducted tests to verify the proportional hazards assumption. The proportional hazards assumption was tested using Schoenfeld residuals. The results of these tests are shown in Figure S1, Supplemental Digital Content, http://links.lww.com/MD/N513, which presents the Schoenfeld residual plots for each covariate (age, distant metastasis, marital status, tumor size, tumor extension, number of tumors, grade, surgery, systemic therapy, and gender). The plots demonstrate that the residuals are randomly scattered around zero without any systematic patterns, indicating no significant violation of the proportional hazards assumption for any of the covariates. The global test for the proportional hazards assumption resulted in a *P* value of .265, indicating no significant violation. The results of these tests provide confidence in the appropriateness of the CoxPH model for our dataset.

### 3.4. Model comparisons

**Figure 3.**
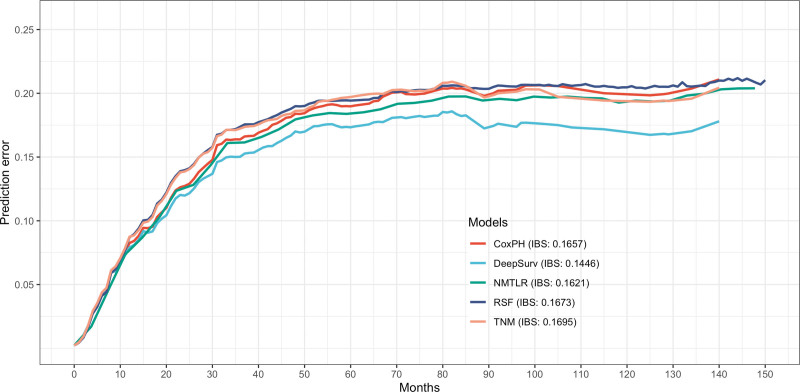
Prediction error curve. As a benchmark, a useful model will have a Brier score below 0.25.

In the test dataset, ML models outperformed the standard CoxPH (0.7349) and TNM model (0.6759) in terms of C-index (DeepSurv: 0.7653; NMLTR: 0.7445; RSF: 0.7403); among those models, DeepSurv achieved the highest C-index value (Table 3). The performance of the model in the training set (DeepSurv: 0.7749; NMLTR: 0.7504; RSF: 0.7590; CoxPH: 0.7370; TNM: 0.6903) and in the test set showed consistent trends and little difference in results, which indicates that the models are not overfitting. The IBS of the 5 models were 0.1657 (CoxPH), 0.1446 (DeepSurv), 0.1621 (NMTLR), 0.1673 (RSF), and 0.1695 (TNM) (Fig. 3).

**Table 3:** Performance of 5 survival models.

	C-index		IBS	3-year AUC	5-year AUC
	Train*	Test*			
CoxPH	0.7370	0.7349	0.1657	0.7713	0.7417
DeepSurv	**0.7749**	**0.7653**	**0.1446**	**0.8014**	**0.7845**
NMLTR	0.7504	0.7445	0.1621	0.7836	0.7645
RSF	0.7590	0.7403	0.1673	0.7828	0.7828
TNM	0.6903	0.6759	0.1695	0.7328	0.7218

CoxPH = standard cox proportional hazards, IBS = Integrated Brier Score, NMLTR = neural multi-task logistic regression, RSF = random survival forest.

* C-index in train and test dataset were calculated separately, and other metrics were calculated in the test set.

**Figure 4.**
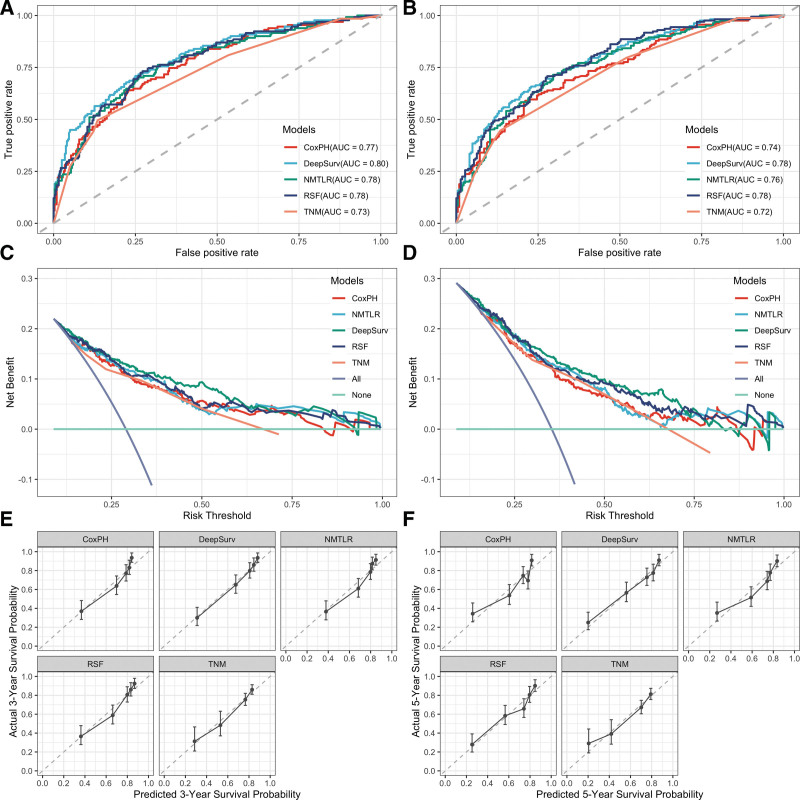
The receiver operating curves (ROC), decision curve analysis (DCA), and calibration curves for 3- and 5-year survival predictions. ROC curves for (A) 3- and (B) 5-year survival predictions. DCA for (C) 3- and (D) 5-year survival predictions. calibration curves for (E) 3- and (F) 5-year survival predictions.

Figure 4A and B shows the discrimination performance of models for 3- and 5-year OS within the test datasets. At both time points, ML models showed a better degree of discrimination (3-year AUC: 0.78–0.80, 5-year AUC: 0.76–0.78), and the traditional CoxPH model (3-year: 0.77, 5-year: 0.74) also had a greater AUC than the TNM model (3-year: 0.73, 5-year: 0.72). The clinical utility of our models was further assessed using DCA, as shown in Figure 4C and D. These curves show that decisions made using the ML and CoxPH models are significantly superior to those made by the TNM model for the thresholds relevant to clinical practice. Among these models, the DeepSurv model showed the best result at an overall level. Calibration curves for 3- and 5-year survival probability estimates revealed an acceptable model calibration, with a good correlation between the survival probability estimates from models and those derived from Kaplan–Meier estimates (Fig. 4E and F).

### 3.5. Risk stratification

**Figure 5.**
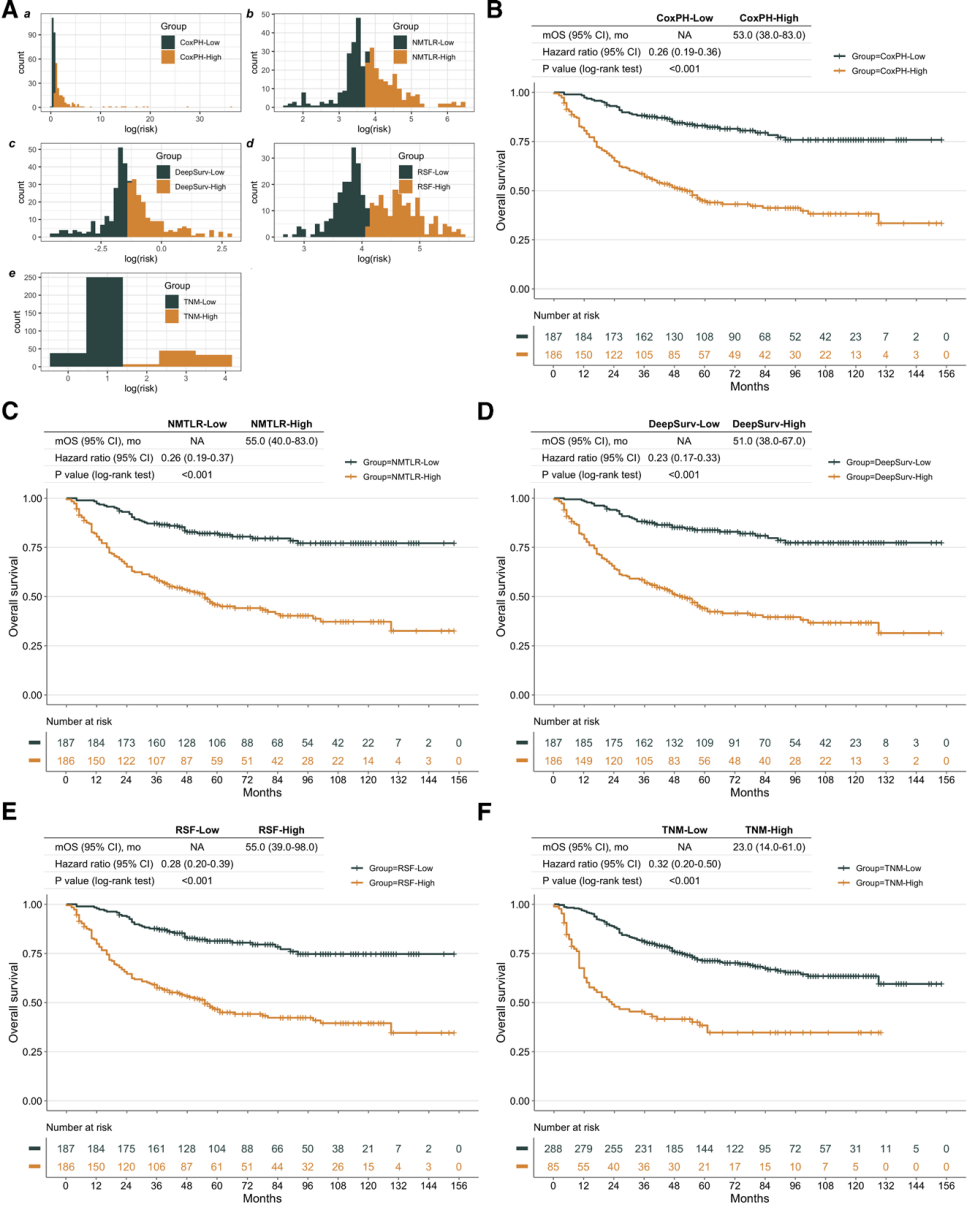
Kaplan–Meier (KM) curves of osteosarcoma patients with different risks stratified by developed models. (A. a–e) According to the risk value generated by the model for all patients, the patients were divided into high- and low-risk groups based on the median risk value. The KM curves of patients grouped by different models are shown in (B–F), respectively.

As shown in Figure 5, all the models showed good performance when used to define high-risk groups (log-rank test *P* < .01), and the high-risk group identified by the DeepSurv model achieved a lower median survival time and higher HR value (reciprocal of low-risk group’s HR value) compared with other models. It is worth noting that because the TNM model only included the Stage feature and the median value of Stage was in Stage IIB, the TNM model only stratified 85 patients with Stage III and IV into the high-risk group.

### 3.6. Treatment recommendation

**Figure 6.**
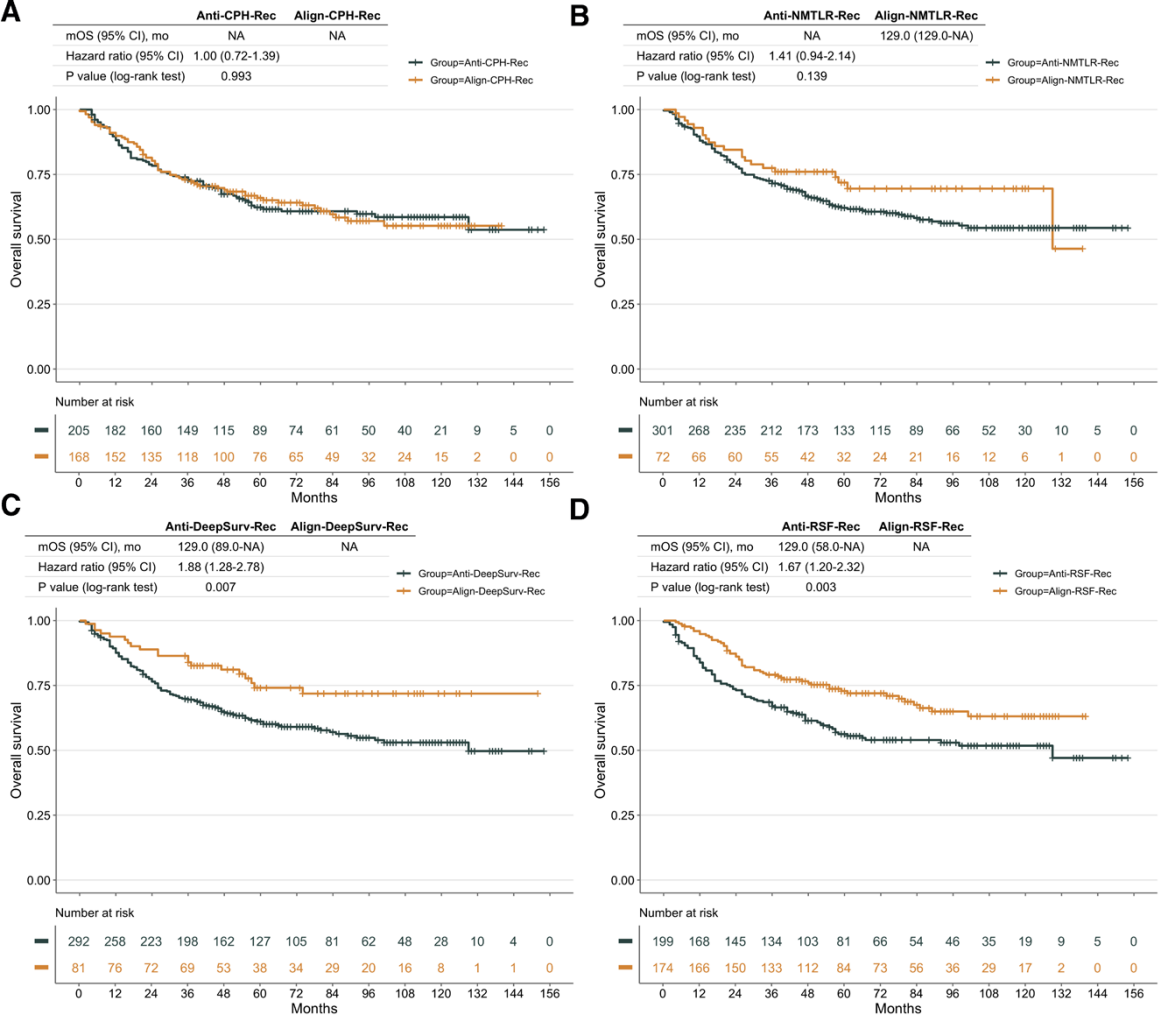
Survival outcomes of the treatment recommendations made by models for chemotherapy in the test dataset. The results are presented for Cox proportional hazards (CoxPH) (A), neural network multitask logistic regression (N-MLTR) (B), DeepSurv (C), and random survival forest (RSF) (D) models. A significant recommendation benefit is seen for patients receiving the chemotherapy treatment recommended by the DeepSurv and RSF models (C, D).

The survival curves of the Align- and Anti-groups were significantly different in the DeepSurv and RSF models (log-rank test *P* < .05, Fig. 6), and patients who didn’t align the treatment option recommended by the DeepSurv model have a higher risk of death than those who violated the RSF recommendations (HR of DeepSurv: 1.88, RSF: 1.67, Fig. 6). Since the CoxPH model recommended preoperative chemotherapy (lower HR value, Table 2) for all patients, the patients receiving preoperative chemotherapy were stratified into the Align- group, and the other patients were stratified into the Anti-group. Although patients who received preoperative chemotherapy had significantly longer survival times than those who did not (Table 2), there was no significant difference in survival time compared to all other patients (Fig. 6).

### 3.7. Feature importance

**Figure 7.**
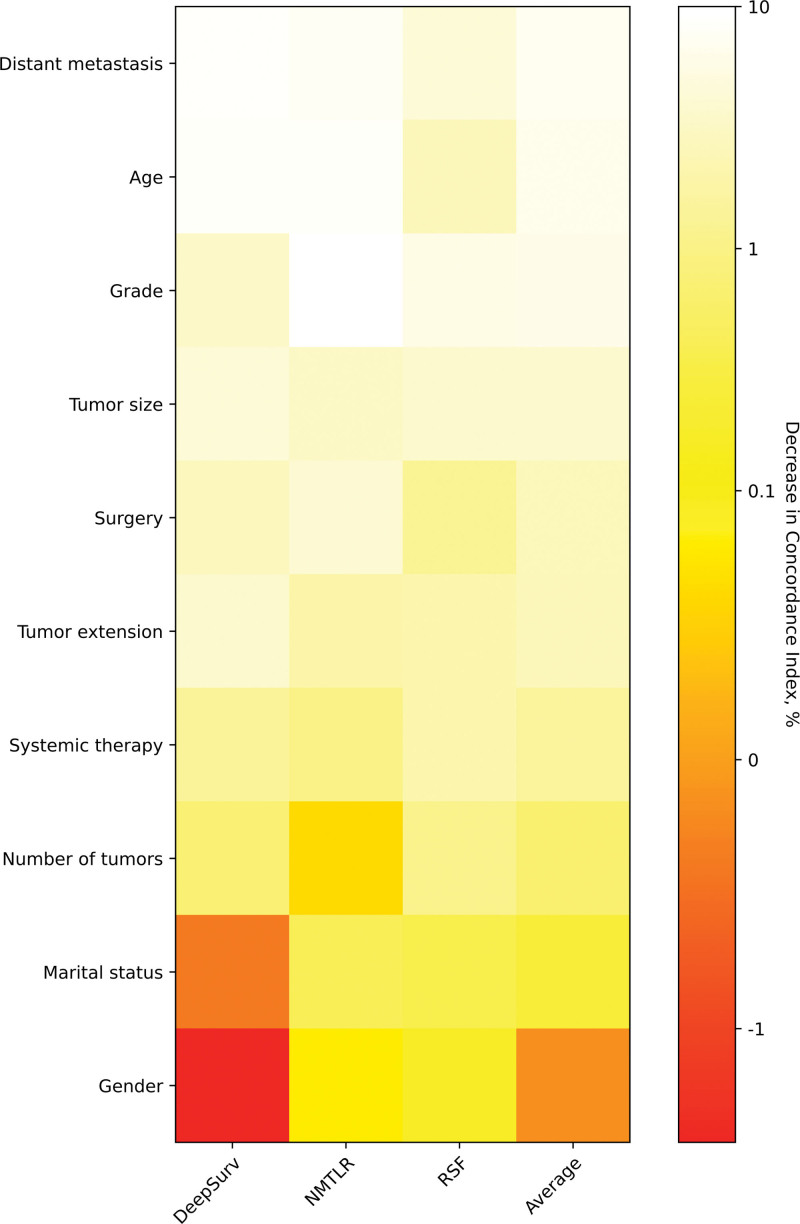
Heatmap of feature importance for DeepSurv, neural network multi-task logistic regression (N-MLTR), and random survival forest (RSF) models.

Assessing the c-index reduction of each feature after random value replacement (Fig. 7) identified the importance of variables to the performance of models for predicting prognosis. Seven out of 10 features contributed to an average 1% decrease in the C-index, namely, distant metastasis, age, grade, tumor size, surgery, tumor extension, and systemic therapy.

### 3.8. Algorithm deployment

**Figure 8.**
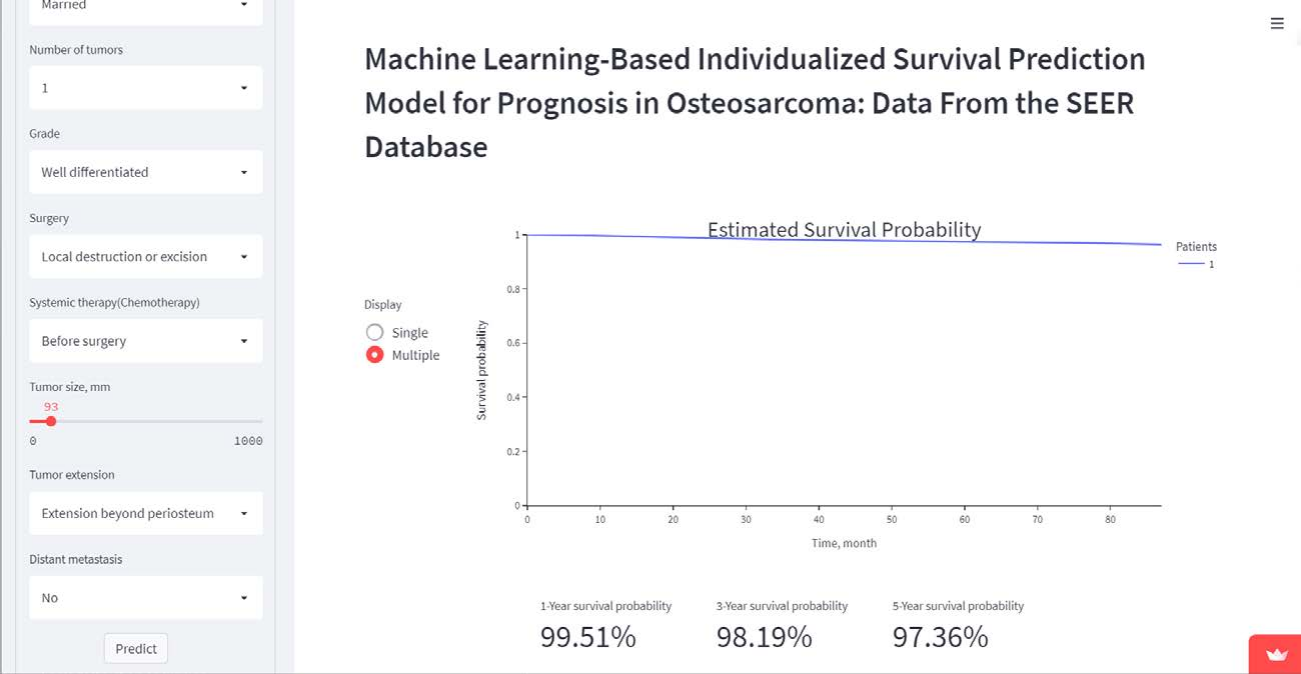
Screenshot of the interactive web page.

Based on the optimal performance DeepSurv model, we built an interactable web application to predict patient survival based on the clinical information provided by the user, which can be accessed at https://survivalofosteosarcoma.streamlit.app/ (Fig. 8).

## 4. Discussion

As treatment regimens of osteosarcoma become more diverse and complex, accurately predicting survival and thus guiding personalized treatment becomes more challenging, so a more integrated approach is needed. In the present study, we first conducted cox regression analyses to identify the risk factors for the osteosarcoma prognosis. We then trained 3 ML models and compared them with the traditional CoxPH model and TNM staging systems. The ML models showed advantages under several evaluation metrics, which provided a new direction for the assessment and treatment of osteosarcoma.

The advantage of the nomogram over the TNM staging system in terms of discrimination is expected because the nomogram model incorporates more variables. Clinical staging systems, such as the AJCC TNM classification and Enneking’s system, identify risk groups based on the clinical characteristics of the tumor (tumor grade, size, metastasis, etc.), have been shown to correlate with the prognosis of osteosarcoma and are widely used in clinical practice.^[34]^ In addition, many other factors have also been reported to be associated with survival, such as patient age, tumor site, type of surgery, and response to chemotherapy.^[34]^ Kim developed a nomogram model based on tumor and clinical characteristics to predict the 5-year probability of metastasis in non-metastatic osteosarcoma of the extremities, with better discrimination than the AJCC TNM staging system (C-index 0.78 vs 0.68; *P* = .02).^[34]^ Beyond clinical factors, more in-depth studies of the underlying mechanism of osteosarcoma have yielded additional serological^[15]^ and molecular^[35,36]^ markers correlated with survival. The nomogram model has been widely used in the construction of prognostic models, with the advantage of fitting multiple variables and providing graphical visualization to predict individual prognosis. However, the nomogram is based on the cox regression and its weakness in ignoring non-linear relationships between variables is evident.

ML algorithms have made significant strides in the field of survival analysis, each technology offering unique advantages while addressing inherent challenges. Traditional support vector machines excel in handling nonlinear relationships and mitigating overfitting, yet they entail substantial computational demands and lack interpretability. Tree-based methods such as decision trees and survival trees provide excellent interpretability but are prone to overfitting. Ensemble learning techniques like bagging, boosting, and RSF have been introduced to enhance predictive capabilities and alleviate these limitations. However, with the increase in data volume and computational power, deep learning and neural networks, composed of multiple layers of neurons, demonstrate clear advantages over traditional linear models or decision tree-based models by learning complex nonlinear relationships in data, particularly evident in addressing intricate problems such as speech recognition, natural language processing, and computer vision.

Three machine learning models were compared, and the results showed that DeepSurv outperforms other models in terms of differentiation, calibration, and clinical benefits, suggesting that it is a powerful tool for predicting prognosis in osteosarcoma. The DeepSurv approach, proposed by Katzman^[22]^ in 2018, offers a new direction for time-to-event prediction. Extending the neural network to the Cox model gives the model the ability to fit non-linear relationships between features, greatly improving the performance of the model. Subsequent studies have also successfully demonstrated the superiority of this method for applications in the medical field.^[28,37]^ In addition, the ability to generate personalized treatment recommendations for a certain patient is also a highlight of the DeepSurv method,^[22]^ which can make optimal treatment recommendations for patients based on the non-linear relationships fitted between patients’ complex clinical features. Howard et al^[30]^ applied this method to a treatment recommendation system for head and neck cancer and observed significant benefits for patients who align the recommendations. In this study, we observed the same results, with patients who aligned the DeepSurv recommended treatment regimen being more likely to have a longer survival time (Fig. 6).

The application of deep learning methods in survival prediction has the additional advantage of flexibility in fitting high-dimensional data. Deep learning models have shown excellent performance on complex, high-dimensional data and have even surpassed humans on some specific prediction tasks. There is also tremendous potential for deep learning in biology and medicine, commonly in medical image classification,^[38]^ brain image segmentation,^[39]^ DNA/RNA binding analysis,^[40]^ epigenome prediction,^[41]^ etc. Extending deep learning to survival analysis methods can significantly broaden the range of applications for time-to-event prediction tasks. Prognosis prediction for patients with tumors is not limited to the use of structured clinical data, but rather to the use of high-dimensional data such as imaging and genetic data, or the use of multi-model fusion methods.

Besides the above-mentioned strengths, there are a couple of limitations that should be mentioned. Firstly, although we have incorporated all prognosis-related features from the SEER database as far as possible, some known prognostic factors have not been considered, which may lead to sub-optimal model performance. Secondly, the model was not validated with external data. Although we used data splitting and cross-validation measures to maximize the generalization of the model, validating the model with external data is still the most straightforward way to verify model generalization. Thirdly, the SEER database, while comprehensive, may have inherent biases and missing data, which could affect the robustness of the model. Future studies should aim to use more diverse datasets to ensure the model’s applicability across different populations. Finally, while our models have demonstrated improved performance over traditional methods, they remain computationally intensive and require significant resources for training and validation. Future research should focus on optimizing these models to be more efficient and accessible for clinical use, ensuring that the benefits of machine learning can be more widely realized in clinical practice.

In conclusion, this study evaluated the application of ML models based on survival analysis to predict the prognosis of patients with osteosarcoma. ML models demonstrated superior performance over traditional CoxPH and TNM models, with DeepSurv displaying optimal results and being developed as a web calculator for clinical use. Future research must address specific elements, such as prognostic biochemical markers and medical imaging data, to support the expanded use of these models for tailored treatment planning and monitoring in orthopedic oncology clinics.

## Author contributions

**Conceptualization:** Ping Cao, Xi Xiang, Weiyi Cheng, Hongjing Li.

**Data curation:** Yixin Dun.

**Formal analysis:** Ping Cao, Weiyi Cheng.

**Funding acquisition:** Hongjing Li.

**Investigation:** Yixin Dun, Weiyi Cheng.

**Methodology:** Ping Cao, Daqing Wang, Weiyi Cheng.

**Resources:** Yixin Dun.

**Software:** Xi Xiang, Lizhao Yan.

**Supervision:** Lizhao Yan, Hongjing Li.

**Validation:** Xi Xiang, Lizhao Yan.

**Visualization:** Ping Cao, Daqing Wang.

**Writing – original draft:** Ping Cao, Xi Xiang, Weiyi Cheng.

**Writing – review & editing:** Ping Cao, Weiyi Cheng, Lizhao Yan.

## Supplementary Material

**Figure:**